# ﻿Taxonomic notes on the little-known genus *Metahelotella* Kirejtshuk (Coleoptera, Cucujoidea, Helotidae) from southwestern China, with description of a new species and two new records

**DOI:** 10.3897/zookeys.1258.171034

**Published:** 2025-11-05

**Authors:** Yu-Zhou Huang, Tian-Xuan Gu

**Affiliations:** 1 College of Plant Protection, Hunan Agricultural University, Changsha 410128, China Hunan Agricultural University Changsha China; 2 Daxuecheng North Road, Chongqing 400053, China unaffiliated Chongqing China

**Keywords:** Cucujiformia, identification key, morphology, new record, new taxon, Oriental region, taxonomy

## Abstract

*Metahelotella alatonotata***sp. nov.** is described from Yunnan, China. Habitus and diagnostic characters of the new species are illustrated. *Metahelotella semifulva* (Ritsema, 1881) and *Metahelotella immaculata* (Ritsema, 1891) are newly recorded from China. A distribution map of the genus *Metahelotella* in Indochina and southwestern China and a taxonomic key to all species are provided.

## ﻿Introduction

Helotidae Chapuis, 1876 is a small and unique family of beetles, whose members are characterized by the following characters: body subflattened, with a complete discrimen and katepisternal suture on the metaventrite; and metacoxae excavate, extending laterally to the elytral epipleura (Kirejtshuk 2000). In 2000, Kirejtshuk revised the previously monogeneric family and divided its species into five genera: *Helota* Maclay, 1825, *Neohelota* Ohta, 1929, *Metahelotella* Kirejtshuk, 2000, *Afrohelotina* Kirejtshuk, 2000, and *Scrophohelota* Kirejtshuk, 2000.

In Asia, the majority of recorded Helotidae species belong to *Helota* and *Neohelota* (Lee 2009a; Lee & Votruba 2014). The genus *Metahelotella* contains only six species, all from the Oriental region. This genus was established by Kirejtshuk (2000) with six species originally placed in the type genus, *Helota*. Later, Lee (2009b) placed the names *Helota fulvitarsis* Ritsema, 1889 and *H. difficilis* Ritsema, 1891 in synonymy with *H. semifulva* Ritsema, 1881, the type species of the genus *Metahelotella*, and described two new species: *Metahelotella schawalleri* Lee, 2009 from Vietnam and *M. sprecherae* Lee, 2009 from China, the latter being the sole Chinese representative of the genus. Subsequently, a fossil species, *Metahelotella monochromata* Liu, Ślipiński, Ren & Pang, 2019, described from Myanmar Cretaceous (Burmese) amber (Liu et al. 2019) was used as a type species for the newly proposed genus *Mesohelotopsis* Kirejtshuk, 2025 (Kirejtshuk 2025). Since Lee’s revision, no additional extant species have been described (Lee 2009b).

The species of the genus *Metahelotella* can be easily distinguished from other Asian Helotidae by their glossy elytral surface that lacks yellow tubercles. Additionally, *Metahelotella* has a color pattern similar to that of some species of the African genera *Afrohelotina* and *Scrophohelota*, but can be distinguished from the former by its smooth elytral surface that lacks costae or tubercles and from the latter by its transverse head and the sexual dimorphism on the protibiae (Kirejtshuk 2000; Lee 2009b).

In this paper, a new species of *Metahelotella* is described from Yunnan, China. Furthermore, *M. semifulva* and *M. immaculata* are recorded from China for the first time.

## ﻿Material and methods

Specimens examined in this study are deposited in the following collections:

**CYZH** Private Collection of Yu-Zhou Huang, Changsha, Hunan;

**IZCAS** Institute of Zoology, Chinese Academy of Sciences, Beijing, China;

**SYSU** Sun Yat-sen University, Guangzhou, China.

Specimens were softened in distilled water and examined under a stereomicroscope. The habitus images were taken using a Sony ILCE-7CII camera equipped with a Laowa FF 100 mm f/2.8L Macro 2:1 Lens, and the character images were taken with a Laowa 25 mm f2.8 2.5-5× Ultra Macro Lens. A SHDM-150 dome light source was used during imaging. Image stacking was performed with Helicon Focus ver. 6.7.1. All images were modified and grouped into plates using Adobe Photoshop CC 2019.

Measurements and the abbreviations used in descriptions are as follows: body length (**BL**): length between the apex of the clypeus and the elytral apex along the mid-line; head length (**HL**): length between the apex of the clypeus and the anterior margin of the pronotum along the mid-line; head width (**HW**): maximum width of head including eyes; pronotum length (**PL**): length of the pronotum along the mid-line; pronotum width (**PW**): maximum width of the pronotum; elytra length (**EL**): length from anterior margin to apices of the elytra; elytra width (**EW**): widest part of both elytron combined.

Distribution information was compiled from Lee (2009b) and the label data of the specimens examined in this study. Morphological terminology follows Kirejtshuk (2000) and Lee (2009b).

## ﻿Results

### ﻿Genus Metahelotella

Taxon classificationAnimaliaColeopteraHelotidae

Kirejtshuk, 2000

3D7448BC-2E32-5C8C-A679-7AB5CB9170CD

Metahelotella Kirejtshuk, 2000: 30.

#### Type species.

*Helota semifulva* Ritsema, 1881: 80, by original designation.

### ﻿ Metahelotella alatonotata sp. nov.

Taxon classificationAnimaliaColeopteraHelotidae

13FBBC52-F149-5237-9F42-2F6DA57AC31E

https://zoobank.org/1FAF9654-DAE3-4A45-B745-BE393C8ADAA9

Figs 1A–G, 2A–H

#### Type materials.

***Holotype*: China** • ♂; Yunnan, Dehong, Yingjiang County, Mangyun Village; 2023.V–VI; local collector leg.; SYSU. ***Paratype*: China** • 1♀; Yunnan, Gaoligong Mountains, Baihualing; 2019.V.22; local collector leg.; SYSU.

#### Differential diagnosis.

This species is similar to *Metahelotella schawalleri* Lee, 2009, but can be easily distinguished by the combination of the following characters: elytral markings larger, not reaching suture; protibiae with green only at basal one-third; apical notch of tegmen deeper (Fig. 2A); penis broader at the apex and more distinctly concave at mesal margin (Fig. 2B) and apical margin of abdominal ventrite V slightly sinuate (Fig. 2D). This species is also similar to *M. semifulva* (Ritsema, 1881), but can be distinguished by the different elytral color patterns and male genital structures.

#### Description.

**Figure 1.**
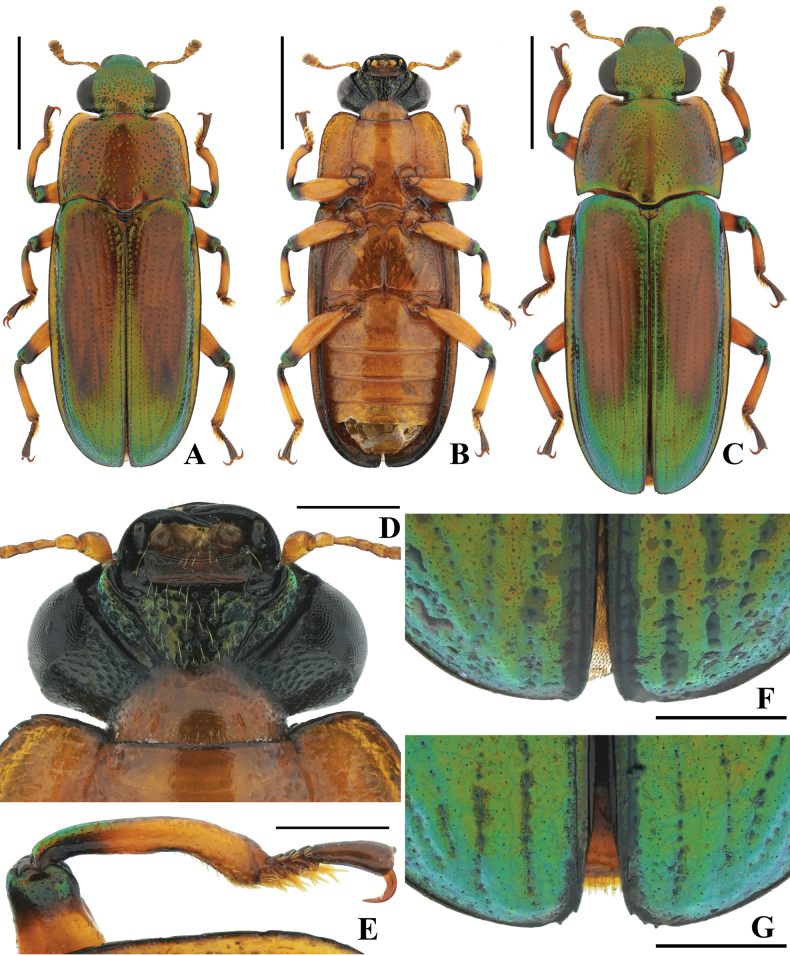
*Metahelotella alatonotata* sp. nov. A. Holotype, ♂, dorsal view; B. Ditto, ventral view; C. Paratype, ♀, dorsal view; D. Holotype, ♂, mentum; E. Ditto, protibia; F. Ditto, teeth on elytral apices; G. Paratype, ♀, teeth on elytral apices. Scale bars: 2 mm (A, B, C); 0.5 mm (D, E, F, G).

**Male.** Body (Fig. 1A, B) flat, surfaces shiny, sides nearly parallel. Eyes black; ventral surface, antennae and legs generally yellowish brown; head, apices of femora and bases of tibiae metallic green with a brownish sheen; tarsi reddish brown; pronotum yellowish brown with a greenish sheen; lateral margin of pronotum and elytra blackish brown; each elytron with one brownish markings from striae 1 to striae 8, reaching apical one-quarter but not reaching the basal margin; remaining areas yellowish green with metallic reflection.

**Figure 2.**
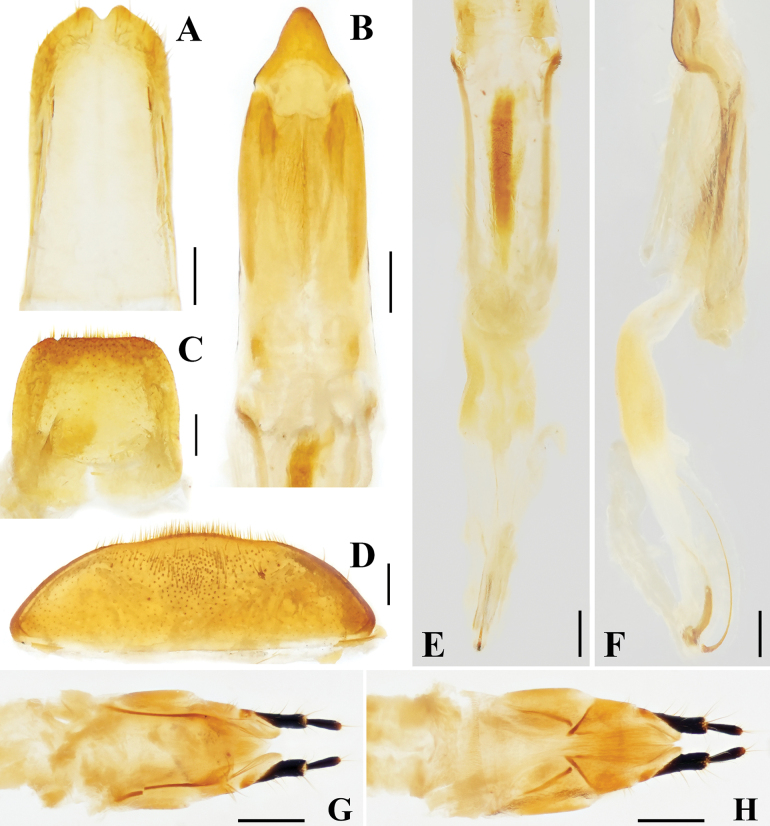
Diagnostic characters of *Metahelotella alatonotata* sp. nov. A. Holotype, ♂, tegmen; B. Ditto, penis; C. Ditto, abdominal tergite VIII; D. Ditto, abdominal ventrite V; E. Ditto, internal sac, dorsal view; F. Ditto, internal sac, lateral view; G. Paratype, ♀, ovipositor, dorsal view; H. Ditto, ventral view. Scale bars: 0.2 mm.

***Head*** triangular, distinctly constricted behind eyes; anterior margin densely covered by tiny punctures and remaining dorsal surface randomly punctate; ventral surface (Fig. 1D) densely punctured on gular area, with even pubescence coverage, and genal area with dense elliptical punctures. Mentum (Fig. 1D) transverse, nearly rectangular, with anterior margin concave. Antenna with 11 antennomeres, including a 3-segmented club.

***Thorax*.** Pronotum nearly trapezoidal, widest across posterior one-third; pronotal disc punctate, weakly convex, punctures denser and larger laterally; anterior angles obtuse, moderately protruding anteriad, posterior angles acute; anterior margin convex, slightly concave at middle; lateral margins weakly serrated, posterior margin distinctly bisinuate. Prothoracic hypomera with sparse punctures. Prosternum, meso- and metaventrites smooth, without punctures at middle. Scutellar shield glabrous, nearly semicircular, lateral margins truncate.

***Elytra*** oblong, widest at basal two-fifths, parallel-sided at basal three-quarters, then gradually narrowing, with apices rounded; each elytron with 10 punctured striae, intervals flat, evenly covered with micro-punctures; lateral margins weakly bordered, apex of each elytron with 1–2 obscure notches (Fig. 1F); epipleura wide, without punctures.

***Legs*.** Femora surfaces smooth, weakly dilated. Protibiae (Fig. 1E) curved, inner side notched and clothed with sparse pubescence on apical one-third. Tarsi simple, tarsomeres I–IV clothed with dense pubescence on ventral surface, tarsomere V longer than the combined length of tarsomeres I–IV.

***Abdomen*** slightly convergent from abdominal ventrite I towards apex; abdominal ventrite V (Fig. 2D) with sparse short setae at middle; apical margin slightly sinuate, with dense short setae that are densest at the apex. Abdominal tergite VIII (Fig. 2C) subquadrate, antero-lateral angles rounded; apical margin truncate, with long setae.

***Male genitalia*.** Penis (Fig. 2B) elongate, with rounded lateral margins, narrowing towards the rounded apex, apical orifice elongate, elliptical (from basal fourth to apical fourth), with long subparallel lobes. Tegmen (Fig. 2A) slender, apical margin with sparse apical setae and a relatively deep notch at middle, basal margin truncate. Internal sac (Fig. 2E, F) with four sclerites basally, one dorsal sclerite long and slender, strongly curved, hooked at base, one ventral sclerite oblong, two paramedian sclerites short, basally expanded.

**Measurements (in mm).**BL 8.02, HL 1.22, HW 1.79, PL 1.86, PW 2.55, EL 5.30, EW 3.02, HL/HW 0.68, PL/PW 0.73, EL/EW 1.74.

**Female** (Figs 1C, G, 2G, H). Similar to male but with body larger; protibiae straight, without notches; and elytral apices (Fig. 1G) with 2–3 distinct teeth. Ovipositor short; paraproct and proximal gonocoxite moderately sclerotized; and distal gonocoxite and gonostylus elongated, slender, and well sclerotized (Fig. 2G, H).

**Measurements (in mm).**BL 8.33, HL 1.28, HW 1.84, PL 1.88, PW 2.64, EL 5.45, EW 3.03, HL/HW 0.69, PL/PW 0.71, EL/EW 1.79.

#### Etymology.

The specific epithet is derived from the Latin words *alatus* and *notatus*, referring to its wing-like elytral markings.

#### Distribution.

China: Yunnan (Fig. 5).

### ﻿ Metahelotella semifulva

Taxon classificationAnimaliaColeopteraHelotidae

(Ritsema, 1881)

9A6F4E48-2E69-57DF-A943-73F72AF509EE

Figs 3A–C, 4A, B

Helota semifulva Ritsema, 1881: 80 (type locality: Indonesia, Java); Wegrzynowicz 2000: 403.
Metahelotella semifulva : Kirejtshuk 2000: 30; Lee 2009b: 786.
Helota fulvitarsis Ritsema, 1889: 107; Wegrzynowicz 2000: 397; synonymized by Lee 2009b: 786.
Metahelotella fulvitarsis : Kirejtshuk 2000: 30.
Helota difficilis Ritsema, 1891: 896; Wegrzynowicz 2000: 396; synonymized by Lee 2009b: 786.
Metahelotella difficilis : Kirejtshuk 2000: 30.

#### Material examined.

**China** • 1♀; Guangxi, Chongzuo City, Longzhou County, Binqiao Village, 22.2810°N, 106.7215°E; 2025.V.2; local collector leg.; CYZH. • 1♂; Yunnan, Dehong Prefecture, Yingjiang County, Mangyun Village; 2025.V; Gui-Chang Liu leg.; CYZH. • 1♂; Xizang, Linzhi City, Medog County; 29.26578°N, 95.15769°E; alt. 1423.92 m; 2024.VII.4; Hong-Bin Liang leg.; IZCAS. **India** • 1♂; Pedong; 1934; collector unknown; CYZH.

#### Diagnosis.

**Figure 3.**
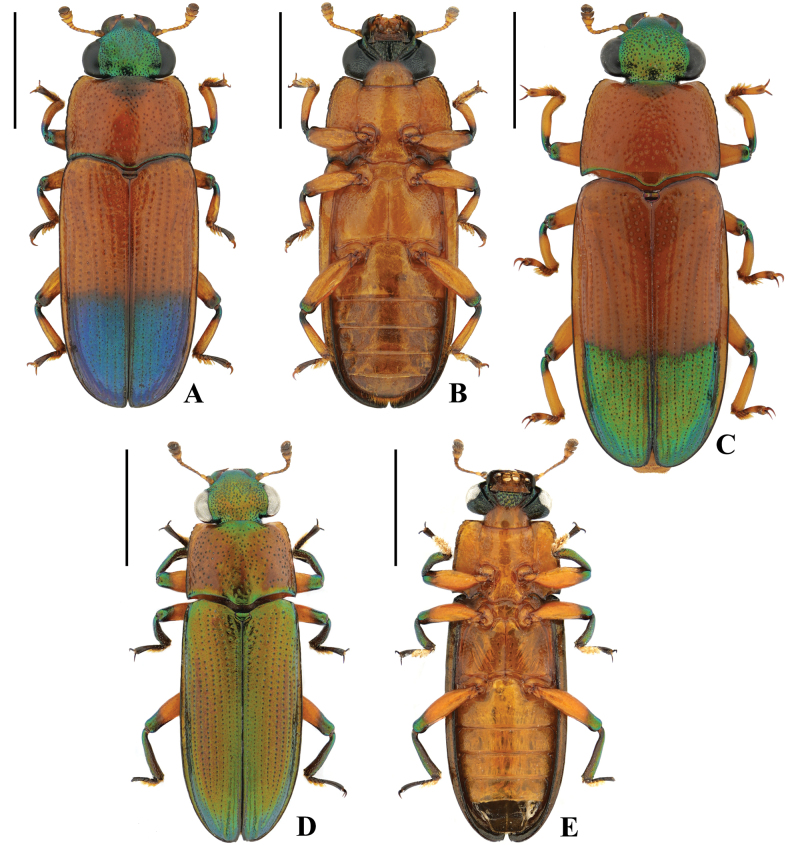
Habitus of *Metahelotella* spp. A. *M. semifulva* from Guangxi, ♀, dorsal view; B. Ditto, ventral view; C. *M. semifulva* from Yunnan, ♂, dorsal view; D. *M. immaculata* from Yunnan, ♂, dorsal view; E. Ditto, ventral view. Scale bars: 2 mm.

Body (Fig. 3A–C) flat, surfaces shiny, sides nearly parallel. Head metallic green; pronotum and basal three-fifths of elytra yellowish brown, remaining dorsal surface greenish blue; apices of femora and bases of tibiae greenish bronze. Protibiae curved in male, inner side notched. Pronotum trapezoidal, with weak crenulations on lateral margins; elytra oblong, widest at basal one-third.

#### Distribution.

China (new country record): Guangxi, Xizang, Yunnan (Fig. 5); India; Indonesia; Laos; Myanmar; Thailand.

#### Remarks.

**Figure 4.**
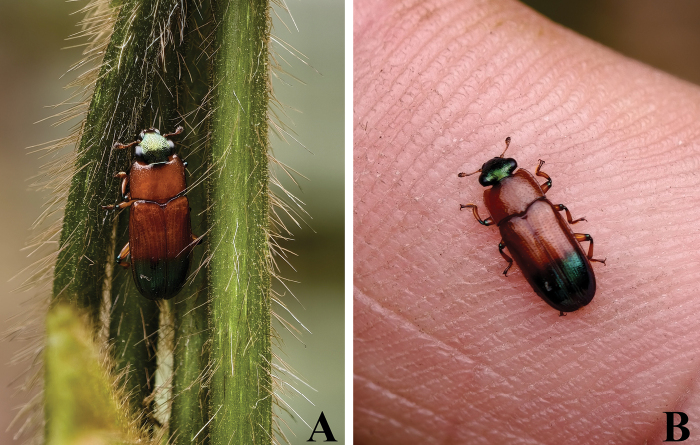
Observation record of *Metahelotella semifulva* (Ritsema, 1881), ♀ A, B. Habitus (Guangxi, Chongzuo, Nonggang Natural Reserve; 2025.III.27; photographed by Yong-Wang Miao).

This species widely occurs in the Oriental region and is reported from China for the first time. In addition, we received an observation record of *M. semifulva* (Ritsema, 1881) from Nonggang Natural Reserve, Guangxi, China (Fig. 4A, B).

### ﻿ Metahelotella immaculata

Taxon classificationAnimaliaColeopteraHelotidae

(Ritsema, 1891)

3EFAE761-E3E6-5711-98C0-12D995360591

Fig. 3D, E

Helota immaculata Ritsema, 1891: 895 (type locality: Myanmar); Wegrzynowicz 2000: 399.
Metahelotella immaculata : Kirejtshuk 2000: 30; Lee 2009b: 790.

#### Material examined.

**China** • 1♂; Yunnan, Dehong Prefecture, Yingjiang County, Mangyun Village; 2025.V; Gui-Chang Liu leg.; CYZH. • 2♀♀; Yunnan, Dehong Prefecture, Yingjiang County, Nongzhang Town, 890 m; 2025.VII.19; Gui-Chang Liu leg.; CYZH. **India** • 1♂, 1♀; Pedong; 1934; collector unknown; CYZH.

#### Diagnosis.

Body (Fig. 3D, E) slender and elongate, sides nearly parallel. Head brownish green; pronotum yellowish brown; elytra brownish bronze with greenish sheen; tibiae and apices of femora greenish bronze. Protibia curved in male. Pronotum broad, subrectangular, lateral margins straight, with weak crenulations near anterior angles; elytra oblong, widest at middle.

#### Distribution.

**Figure 5.**
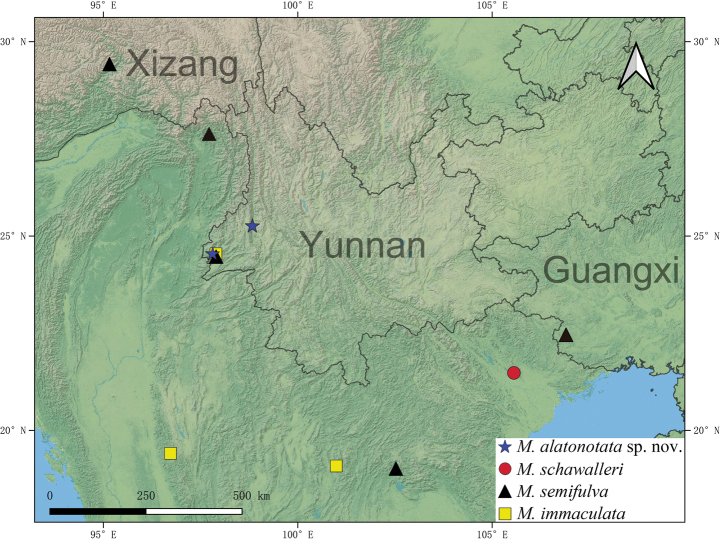
Distribution map of *Metahelotella* spp. in Indochina and southwestern China.

China (new country record): Yunnan (Fig. 5); India; Myanmar; Thailand.

#### Remarks.

The specimens from Yunnan appear morphologically consistent with those from India.

## ﻿Discussion

The distribution of this genus ranges across the Oriental region (Kirejtshuk 2000; Lee 2009b). The new records fill the distribution gap of this genus in southwestern China (Lee 2009b). Nevertheless, as a little-known genus of Helotidae, few observations and records of this genus have been reported from China. Currently, the lack of targeted collection may have led to this situation.

In both Pedong, India and Yingjiang, China, *M. immaculata* (Ritsema, 1891) and *M. semifulva* (Ritsema, 1881) were collected simultaneously, and *M. alatonotata* sp. nov. was also collected in the same village of Yingjiang, indicating that these species share the same habitat and have similar occurrence patterns.

### ﻿Key to species of *Metahelotella* (based on Lee 2009b)

**Table:** 

1	Elytra with distinct yellowish-brown markings	**2**
–	Elytra without distinct yellowish-brown markings	**4**
2	Basal three-fifths of elytra yellowish brown, without greenish sheen (Fig. 3A, C)	***M. semifulva***
–	Sides and basal margin of elytra greenish bronze or yellowish green	**3**
3	Elytral markings reaching suture	***M. schawalleri***
–	Elytral markings not reaching suture (Fig. 1A, C)	***M. alatonotata* sp. nov.**
4	Pronotal color same as elytra	***M. marthae***
–	Pronotal color at least partly different from elytra	**5**
5	Pronotum with a median longitudinal greenish-bronze band	***M. bouchardi***
–	Pronotum without a median longitudinal greenish-bronze band	**6**
6	Pronotum bronze, elytra greenish bronze, tibiae generally yellowish brown	***M. sprecherae***
–	Pronotum yellowish brown, elytra brownish bronze, tibiae generally metallic green (Fig. 3D)	***M. immaculata***

## Supplementary Material

XML Treatment for Metahelotella

XML Treatment for Metahelotella alatonotata

XML Treatment for Metahelotella semifulva

XML Treatment for Metahelotella immaculata

## ﻿Additional information

### Use of AI

No use of AI was reported.

### Author ORCIDs

Yu-Zhou Huang https://orcid.org/0000-0002-0884-1408

Tian-Xuan Gu https://orcid.org/0009-0009-2101-9388

### Data availability

All of the data that support the findings of this study are available in the main text.
